# *Escherichia coli* and their potential transmission of carbapenem and colistin-resistant genes in camels

**DOI:** 10.1186/s12866-024-03215-6

**Published:** 2024-02-24

**Authors:** Marwa youseef, Fatma Karam, Mona Kadry, Mahmoud Elhariri, Rehab Elhelw

**Affiliations:** 1 Department of Microbiology and Immunology, Faculty of Veterinary Medicine, Cairo University, PO Box 12211, Giza, Egypt; 2 Department of Zoonoses, Faculty of Veterinary Medicine, Cairo University, PO Box 12211, Giza, Egypt

**Keywords:** Camel, *E. Coli*, Egypt, *Mcr* genes, *Stx* genes

## Abstract

**Background:**

Camels harbouring multidrug-resistant Gram-negative bacteria are capable of transmitting various microorganisms to humans. This study aimed to determine the distribution of anti-microbial resistance among *Escherichia coli* (*E. coli*) isolated from the feces of apparently healthy camels in Egyptian abattoirs. Additionally, we sought to characterize Shiga toxin-producing *E. coli* (STEC) strains, assess their virulence potential, and investigate the possibility of camels spreading carbapenem- and colistin-resistant *E. coli*.

**Methods:**

121 fecal swaps were collected from camels in different abattoirs in Egypt. Isolation and identification of *E. coli* were performed using conventional culture techniques and biochemical identification. All isolates obtained from the examined samples underwent genotyping through polymerase chain reaction (PCR) of the Shiga toxin-encoding genes (*Stx1* and *Stx2*), the carbapenemase-encoding genes (*bla*_*KPC*_, *bla*_*OXA−48*_, *bla*_*NDM*_, and *bla*_*VIM*_), and the *mcr* genes for *mcr-1* to *mcr-5*.

**Result:**

Bacteriological examination revealed 75 *E. coli* isolates. PCR results revealed that one strain (1.3%) tested positive for *Stx1*, and five (6.6%) were positive for *Stx2*. Among the total 75 strains of *E. coli*, the overall prevalence of carbapenemase-producing *E. coli* was 27, with 7 carrying *bla*_OXA48_, 14 carrying bla_*NDM*_, and 6 carrying bla_*VIM*_. Notably, no strains were positive for *bla*_*KPC*_ but a high prevalence rate of *mcr* genes were detected. *mcr-1, mcr-2, mcr-3, and mcr-4* genes were detected among 3, 2, 21, and 3 strains, respectively.

**Conclusion:**

The results indicate that camels in Egypt may be a primary source of anti-microbial resistance (AMR) *E. coli*, which could potentially be transmitted directly to humans or through the food chain.

**Supplementary Information:**

The online version contains supplementary material available at 10.1186/s12866-024-03215-6.

## Background

Camels play a vital role in the socioeconomic growth of many nations, particularly in Africa and the Middle East [1]. They provide essential resources and services that benefit local communities and economies, such as milk and meat production, transportation, and entertainment, particularly in Egypt’s tourism sector [2].

The impact of climate change and the rise in drought conditions have shifted livestock preferences in various regions worldwide, leading to a notable increase in the abundance of camels [3].

Camels in sub-Saharan Africa serve as a reservoir for various potentially zoonotic diseases and pathogens [4]. Carcass microbiological contamination primarily occurs during processing and handling stages, such as skinning, evisceration, preparation, storage, and distribution at abattoirs and retail shops [5].

*Escherichia coli*, or *E. coli*, is a rod-shaped bacterium in the intestinal tracts of humans and warm-blooded animals. While many strains of *E. coli* are harmless and coexist naturally, certain strains, such as Shiga toxin-*E. coli* (STEC), can result in foodborne illnesses. Some *E. coli* strains can also cause infections in the urinary and respiratory systems and other diseases [6].

In contrast, STEC in camels, capable of causing gastrointestinal illnesses such as non-bloody or bloody diarrhoea, haemorrhagic colitis (HC), and haemolytic uremic syndrome (HUS), has been rarely documented. However, STEC is responsible for approximately 2,801,000 cases of acute illnesses each year, posing a substantial global health burden [7].

*E. coli* produces numerous highly virulent genes, with Shiga toxin-producing *E. coli* (STEC) being the most significant serotype regarding public health toxins. The virulence factors of STEC are primarily derived from Shiga toxin genes (*Stx1* and *Stx2*), which play a significant role in the manifestation of clinical symptoms [8]. Furthermore, *Stx1* and *Stx2* can have sequence variants, and a single STEC bacterium can produce multiple variants of these toxins [9].

The primary mode of transmission of STEC to humans is through consuming contaminated foods, including raw or undercooked ground meat products and unpasteurized milk. Additionally, cross-contamination during food preparation is another significant mode. Hand-to-mouth transfer involving direct contact with farm animals is also identified as a substantial transmission mode [10].

Antibiotic resistance (AMR) poses a significant threat to human and animal health, making the treatment of bacterial infections increasingly challenging. One of the essential AMR mechanisms in the Enterobacteriaceae family involves the production of extended-spectrum β-lactamases (ESBLs) and metallo-β-lactamases (MBLs), which can inactivate a wide range of antibiotics, including carbapenems, considered last-line therapies [11].

Gram-negative bacteria can resist antibiotics in several ways, such as by producing enzymes that destroy antibiotics, making it harder for antibiotics to enter the cell, or changing the cell membrane’s structure to prevent antibiotic penetration. These changes have been seen in many bacteria resistant to multiple drugs [12].

Carbapenems, important in human medicine as broad-spectrum beta-lactam antibiotics, are considered the last-line therapies for severe infections. The five most crucial carbapenemase enzymes are KPC, NDM, IMP, VIM, and OXA. These enzymes can break down carbapenems, conferring antibiotic resistance [13].

*E. coli* can resist many different types of antibiotics; the most common is beta-lactam resistance, including cephalosporins, aminoglycosides, and tetracyclines. *E. coli* achieves this by producing enzymes called beta-lactamases associated with genes such as *bla*_*TEM*_ and *bla*_*CTX*_, which code for beta-lactamases [14].

Colistin, a potent antibiotic used in treating severe infections and often considered a last-resort antibiotic, faces the challenge of resistance, which can spread to other bacteria through mobile genetic elements, rapidly spreading this resistance within bacterial populations. A plasmid known as *mcr-1*, capable of transmitting colistin resistance to other bacteria [15, 16], can also be found on plasmids carrying other antibiotic-resistance genes, including those encoding carbapenemases and extended-spectrum beta-lactamases [17]. Many studies have demonstrated that using colistin as an antibiotic growth promoter (AGP) in livestock contributes to the emergence and spread of plasmid genes, conferring resistance to polymyxins, including colistin itself [18].

This study aimed to update our understanding of the prevalence of *E. coli* bacteria in camels in Egypt, characterize the strains of *E. coli* causing STEC infections, assess the potential of camels to spread *E. coli* bacteria resistant to carbapenem and colistin antibiotics, and evaluate the potential risk to human and animal health arising from the transmission of these strains to the environment.

## Methods

Sample preparation: A total of 121 faecal swaps were collected from camels aged 3–5 years in different abattoirs in Cairo and Giza governorates (Al Waraq and Al Basateen abattoirs) during the period from January 2022 to June 2022. Subsequently, the swabs were placed in 2 ml of sterile saline (0.9% NaCl) and stored in an ice box until transported to the laboratory.

Bacterial isolation and identification: All samples were inoculated into brain-heart infusion broth tubes and incubated at 37 °C for 24 h. A loopful from the previously incubated tubes was streaked on eosin methylene blue agar (EMB) and incubated aerobically at 37 °C for 24–48 h. Suspected colonies were purified through subculture on EMB agar plates and subjected to traditional biochemical tests, including indole, methyl red, Voges-Proskauer, citrate utilization, and urease tests [19]. The isolates were stored at − 20 °C until further molecular analysis.

Molecular detection of virulence and antibiotic resistance genes in *E. coli*: All isolates obtained from the examined samples underwent genotyping using polymerase chain reaction (PCR), according to the protocol described by [20]. The template DNA used consisted of boiled lysates prepared from the isolates. In brief, a loopful of culture was suspended in 100 µl of sterile TE buffer, boiled for 10 min at 100 °C, and centrifuged for 5 min at 6000×g. The extracted DNA was stored at -20 °C until use.

All genomic DNA of the identified *E. coli* strains underwent PCR testing for Shiga toxin-encoding genes (*Stx1* and *Stx2*) using multiplex PCR assays. The target genes, oligonucleotide primer sequences, and the expected product size in different PCR assays are outlined in Table 1.

**Table 1:** List of primer pairs used for the *stx1* and *stx2* genes in this study

Target	Primer sequence (5`→3`)	Band size	Reference
Stx 1	CGA TGT TAC GGT TTG TTA CTGTGA CAG C	244	[47]
	AAT GCC ACG CTT CCC AGA ATT G		
Stx2	CCA TGA CAA CGG ACA GCA GTT	779	[48]
	CCT GTC AAC TGA GCA GCA CTT TG		

To detect the carbapenemase-encoding genes (*bla*_KPC_, *bla*_OXA−48_, *bla*_NDM_, and bla_VIM_), multiplex PCR was performed using specific oligonucleotide primers for detecting bla_KPC_ and bla_NDM_ (Table 2). The PCR mixtures had a total reaction volume of 25 mL. All reaction mixtures were subjected to 30 cycles at 94 °C for 1 min, 55 °C for 1 min, and 72 °C for 2 min. Subsequently, 5 mL of the PCR product was electrophoresed on a 1% agarose gel to determine the size of the product [21]. Uniplex PCR was also conducted using a specific oligonucleotide primer to detect bla_VIM_ (Table 2). The PCR mixtures had a total reaction volume of 25 mL. All reaction mixtures were subjected to 35 cycles at 94 ºC for 30 s, 55 ºC for 30 s, 72ºC for 1 min, and a final elongation at 72 ºC for 10 min. Then, 5 mL of the PCR product was electrophoresed on a 1% agarose gel to determine the size of the product [22].

**Table 2:** Primer sequences used for PCR amplification of carbapenemase encoding genes

Target	Primer sequence (5`→3`)	Band size	Reference
KPC	ATG TCA CTG TAT CGC CGT CT	882	[49]
	TTT TCA GAG CCT TAC TGC CC		
NDM	GGT TTG GCG ATC TGG TTT TC	621	
	CGG AAT GGC TCA TCA CGA TC		
OXA 48	GCTTGATCGCCCTCGATT	283	[50]
	GATTTGCTCCGTGGCCGAAA		
VIM	AGTGGTGAGTATCCGACAG	261	[51]
	ATGAAAGTGCGTGGAGAC		

The plasmid DNA served as the template for PCR. The primer pair used to detect the bla_OXA−48_ gene consisted of bla_OXA−48_ F(5ʹ-GCTTGATCGCCCTCGATT-3ʹ) and bla_OXA−48_ R (3ʹ-GATTTGCTCCGTGGCCGAAA-5ʹ). The thermal cycling process consisted of initial denaturation at 94 °C for 10 min, denaturation at 94 °C for 40 s, annealing at 60 °C for 40 s, extension at 72 °C for 1 min, and a final extension at 72 °C for 7 min. In total, 30 cycles were run. The amplified products were then subjected to gel electrophoresis [22].

Multiplex PCR was also performed using oligonucleotide primers for *mcr-1* to *mcr-5* (Table 3). The PCR conditions included denaturation at 94 °C for 5 min, followed by 25 cycles of denaturation at 94 °C for 30 s, annealing at 58 °C for 90 s, elongation at 72 °C for 60 s, and a final cycle of elongation at 72 °C for 10 min.

**Table 3:** List of primers used for the mcr 1–5 genes

Target	Primer sequence (5`→3`)	Band size	Reference
MCR1	AGTCCGTTTGTTCTTGTGGC	320	[52]
	AGATCCTTGGTCTCGGCTTG		
MCR2	CAAGTGTGTTGGTCGCAGTT	715	
	TCTAGCCCGACAAGCATACC		
MCR3	AAATAAAAATTGTTCCGCTTATG	929	
	AATGGAGATCCCCGTTTTT		
MCR4	TCACTTTCATCACTGCGTTG	1116	
	TTGGTCCATGACTACCAATG		
MCR5	ATGCGGTTGTCTGCATTTATC	1644	
	TCATTGTGGTTGTCCTTTTCTG		

## Results

Isolation and identification of *E. coli* strains:

The bacteriological examination of 121 camels’ faecal swaps showed the presence of 75 *E. coli* isolates (Table 4). Using multiplex PCR for the detection of Shiga toxin-encoding genes (*Stx1* and *Stx2*) (Table 5), one *E. coli* strain (1.3%) tested positive for *Stx1*, and five strains (6.6%) were positive for *Stx2*.

**Table 4:** Occurrence rate of *E-coli* isolates from apparent healthy camels in Egyptian abattoirs

Area of Study	Samples	No. of samples	No. of positive (%)
Al Waraq abattoir	Faecal swaps	86	56(65.11)
Al Basateen abattoir		35	21(60)
Total		121	75 (61.98)

**Table 5:** Molecular detection of Shiga toxin genes (*Stx1* and *Stx2*), the carbapenemase-encoding genes (*bla*_*KPC*_, *bla*_*OXA−48*_, *bla*_*NDM*_, *and bla*_*VIM*_*)* and colistin resistance genes (mcr-*1, mcr-2, mcr-3, mcr-4*, and *mcr-5*) among *E. coli* isolates

Total *E. coli* Isolates	Positive for *Stx1* (%)	Positive for *Stx2* (%)	carbapenemase-encoding genes (%)				Colistin resistance genes (%)				
			*bla*_*KPC*_	*bla*_*OXA−48*_	*bla*_*NDM*_	*bla*_*VIM*_	*Mcr1*	*mcr2*	*mcr3*	*mcr4*	*mcr5*
75	1(1.3)	5 (6.6)	0(0)	7(9.3)	14(18.6)	6(8)	3(4)	2(2.6)	21(28)	3(4)	0(0)
			27(36)				29(38.66)				

Molecular findings of virulence and antibiotic resistance genes in *E. coli*:

In Table 5, the total prevalence of carbapenemase-producing *E. coli* was 27 strains: 7 carrying bla_*OXA−48*_, 14 carrying bla_*NDM*_, and 6 carrying bla_VIM_, while no strain carried bla_KPC_. Additionally, the total prevalence of colistin resistance genes in *E. coli* isolates was 29 strains, with *mcr-1, mcr-2, mcr-3, mcr-4*, and *mcr-5* being 3, 2, 21, 3, and 0, respectively.

## Discussion

Camels are susceptible to several infectious diseases, meaning eating camel meat or coming into contact with camels can pose a significant risk of zoonotic disease transmission [23]. In this study (Table 4), special consideration aligns with Jones et al. [24], who concluded that eating raw camel meat often leads to outbreaks of diarrheagenic *E. coli*, a type of bacteria that can cause diarrhea. This typically occurs due to rough handling procedures during slaughter and transportation. Additionally, many countries worldwide have reported a high incidence of pathogenic *E. coli* strains in fresh camel milk [25].

STEC is a type of bacteria that can cause food poisoning. It is a zoonotic pathogen responsible for mild to severe diarrhea, hemorrhagic colitis (bloody diarrhea), and hemolytic uremic syndrome (HUS) [26]. The distinguishing feature of STEC is the presence of one or more Shiga toxin (*Stx*) genes, which code for proteins that can damage the cells in the lining of the intestines. There are two main types of Shiga toxins: *Stx1* and *Stx2* [27].

Most *E. coli* bacteria live in the intestines of humans and animals without causing any harm. However, some *E. coli* bacteria produce toxins, leading to food poisoning. STEC infections are most common in ruminants. These animals can carry STEC bacteria in their intestines without getting sick. However, these bacteria can be spread to people through food or water contaminated with animal feces [28, 29]. This transmission occurs because the lining of their intestines lacks vascular receptors, preventing the toxins from being absorbed into the bloodstream and transported to other organs. As a result, the STEC bacteria can colonize the large intestine without causing symptoms [30].

The prevalence of Shiga toxin-encoding genes (*Stx1* and *Stx2*) detected in this study was closely consistent with those reported by Erickson and Doyle [31] and Kintz et al. [32]. These studies focused on uncovering the source and transmission of STEC infections in the food chain and humans.

STEC can be transmitted to humans in several ways, including eating undercooked ground beef or other raw foods, such as lettuce, sprouts, or spinach, from manured gardens, drinking contaminated water or unpasteurized milk or juice, coming into direct contact with animal feces, or being infected with STEC [33].

Extended-spectrum beta-lactamase (ESBL)-producing Enterobacteriaceae (ESBL-E) are bacteria equipped with enzymes that can break down a wide range of beta-lactam antibiotics, including penicillin and cephalosporins. However, they are not resistant to carbapenem antibiotics [34]. ESBL-E bacteria pose a serious public health threat as they can be difficult or impossible to treat, leading to more extended hospital stays, more severe illnesses, and even death [35]. Infections caused by ESBL-producing Enterobacteriaceae (ESBL-E) are concerning for various reasons, including increased hospital costs, length of hospital stays, and mortality rates. ESBL-E infections can also be more deadly than other infections, necessitating treatment with last-resort antibiotics like carbapenems [36]. Recently, the effectiveness of carbapenems has been disposed of globally by the emergence of carbapenem-resistant bacteria. The resistance of Enterobacteriaceae to carbapenems includes numerous mechanisms, the most significant being the production of carbapenemases.

In Table 5, the prevalence of carbapenemase-producing *E. coli* was 27 strains: 7 carried bla_*OXA*_, 14 carried bla_*NDM*_, and 6 carried bla_*VIM*_, while no strain carried bla_*KPC*_. This result agrees with Tzouvelekis et al. [37], who concluded that the clinical intake of carbapenems has increased, leading to a rise in the number of pathogen isolates producing carbapenemases. The increased prevalence of bacterial species carrying ESBL genes, as reported worldwide, included community-acquired Escherichia coli isolates with the ability to produce ESBLs [38]. Moreover, this result agrees with Cantón et al. [39], who discussed that KPC, OXA, and NDM involve three of the ‘big five’ carbapenemases associated with nosocomial contagions. The global increase in carbapenemase-producing Enterobacteriaceae (CPE) has led to the overuse of colistin. This overuse has raised concerns about the emergence of colistin resistance (*mcr*) genes in bacteria [40], which are already resistant to many other antibiotics. Additionally, data on colistin-resistant *E. coli* and *mcr* genes in camels are lacking. Given the increasing use of camels for meat and milk production, this is a concern, raising the possibility that camels play a role in transmitting colistin-resistant bacteria to humans.

the present study showed a surprising occurrence of *mcr* genes in *E. coli* isolates where the total prevalence of colistin resistance genes in *E. coli* isolates was 29 (Table 5) and these findings was much higher than those obtained by Rhouma et al., who found no colistin resistance in *E. coli* isolated from camel feces in southern Tunisia [41].

Veterinarians working with camels face a significant challenge because there is no approved anti-microbial to treat bacterial infections in these animals. Anti-microbials approved for ruminants, horses, or other animal species to treat sick camels have proven ineffective due to the unique physiology of camels [42].

In a recent study in Egypt, *Kamel et al.* [43] investigated carbapenem-resistant Gram-negative bacteria isolated from febrile paediatric cancer patients from October 2014 to December 2016. The study revealed that *bla*_*OXA−48*_ was the most ubiquitous carbapenemase gene (58.62%), followed by *bla*_*NDM*_ (27.58%), *bla*_*VIM*_ (10.3%), and *bla*_*KPC*_ (6.89%).

Evidence shows that camels could be a significant source of *mcr* gene contamination for Egypt’s local population and tourists. This is because camels and tourists often come into close contact, potentially spreading resistant bacteria globally.

New plasmid-mediated *mcr* genes have rapidly emerged in the past four years, compromising the therapeutic effectiveness of colistin, a last-resort antibiotic used to treat multidrug-resistant bacterial infections [44].

The *mcr-1* and *mcr-2* genes have engrossed worldwide consideration, heralding the polymyxin gap. However, in this study, the *mcr-3* gene exhibited a prevalence of 28% compared with the mcr-1, mcr-2, mcr-4, and mcr-5 genes, which had prevalence rates of 4%, 2.6%, 45%, and 0%, respectively. These results agree with Yin et al. [45], who examined a colistin-resistant Escherichia coli isolate. The study yielded negative results for *mcr-1* and *mcr-2* and discovered a novel *mcr-3*. They found wide-ranging *mcr-3* between *Enterobacteriaceae* and *Aeromonas* species initiating from clinical infections and environmental specimens across twelve countries on four continents.

*E. coli*, normal inhabitants of the intestines of humans and mammals, potentially represent a significant reservoir of AMR and play a vital role in gaining and propagating AMR mechanisms. Since colistin is widely used in veterinary medicine and is increasing in use in human medicine, it is crucial to continuously monitor the spread of *mcr* genes in both the agricultural and healthcare sectors. This can be achieved by tracking the presence of mobile colistin resistance determinants in colistin-resistant Gram-negative bacteria [46].

## Conclusion

STEC is a significant foodborne zoonotic bacterium, and camels may play a role in transmitting E. *coli*, which resists many antibiotics to humans. These results recommend the need for careful veterinary practice of beta-lactams in the camel industry. For the first time in Egypt, camels could become a source of the *mcr-3* gene. Therefore, the search for the *mcr-3* gene should be immediately encompassed in investigating colistin-resistant Gram-negative bacteria from animals, humans, and the environment.

### Electronic supplementary material

Below is the link to the electronic supplementary material.

Supplementary Material 1

Supplementary Material 2

## Data Availability

No datasets were generated or analysed during the current study.
