# Supplementary material for: Escherichia coli and their potential transmission of carbapenem and colistin-resistant genes in camels
Source: BMC Microbiol. 2024 Feb 24;24:65. doi: 10.1186/s12866-024-03215-6 (PMC10893666; doi:10.1186/s12866-024-03215-6)
Supplement: Supplementary file 1 — Supplementary Material 1 [file 12866_2024_3215_MOESM1_ESM.docx]

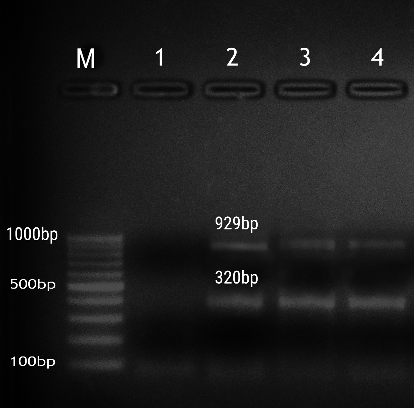


Figure S1: Agarose gel electrophoresis of multiplex PCR showing amplification of mcr-1(320bp) and mcr-3(929bp).

M: DNA marker GeneRuler 100 bp (Thermofisher), lane 1: negative control.


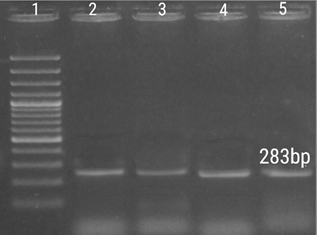


Figure S2: Agarose gel electrophoresis of uniplex PCR showing amplification of bla_OXA48_(283bp), 1: DNA marker GeneRuler 100 bp plus (Thermofisher).


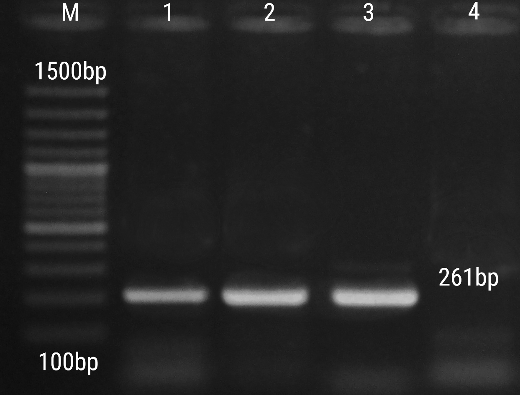


Figure S3: Agarose gel electrophoresis of uniplex PCR showing amplification of bla_VIM_ (261 bp), M: DNA marker GeneRuler 100 bp plus (Thermofisher).


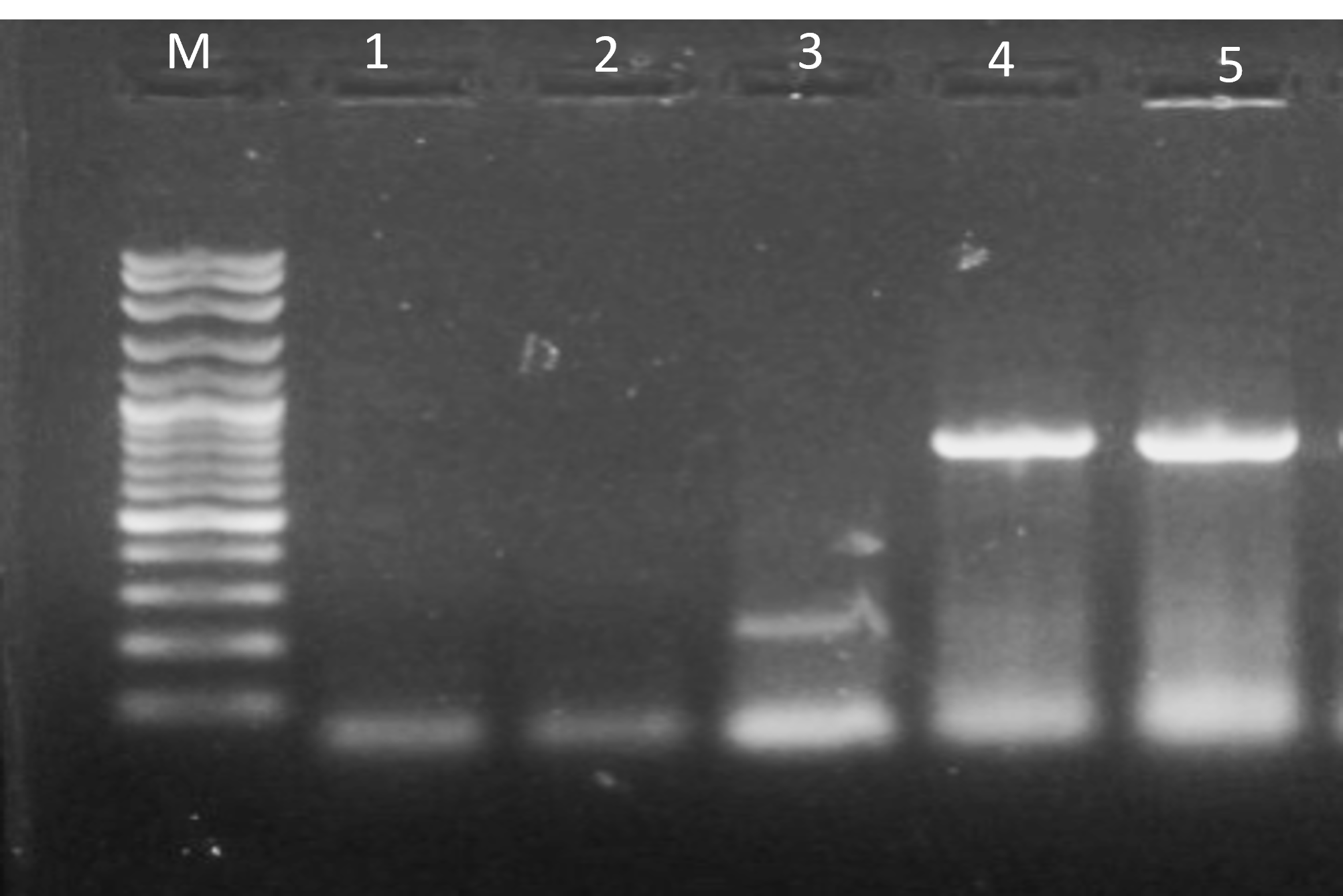


Figure S4: Agarose gel electrophoresis of multiplex PCR showing amplification of Stx1(244bp) and Stx2 (779), M: DNA marker GeneRuler 100 bp plus (Thermofisher).

**Figures:**

**Figure S1:** Agarose gel electrophoresis of multiplex PCR showing amplification of mcr-1(320bp) and mcr-3(929bp).

M: DNA marker GeneRuler 100 bp (Thermofisher), lane 1: negative control.

**Figure S2:** Agarose gel electrophoresis of uniplex PCR showing amplification of bla_OXA48_(283bp), 1: DNA marker GeneRuler 100 bp plus (Thermofisher).

**Figure S3:** Agarose gel electrophoresis of uniplex PCR showing amplification of bla_VIM_ (261 bp), M: DNA marker GeneRuler 100 bp plus (Thermofisher).

**Figure S4:** Agarose gel electrophoresis of multiplex PCR showing amplification of Stx1(244bp) and Stx2 (779), M: DNA marker GeneRuler 100 bp plus (Thermofisher).
