# Supplementary material for: Escherichia coli and their potential transmission of carbapenem and colistin-resistant genes in camels
Source: BMC Microbiol. 2024 Feb 24;24:65. doi: 10.1186/s12866-024-03215-6 (PMC10893666; doi:10.1186/s12866-024-03215-6)
Supplement: Supplementary file 2 — Supplementary Material 2 [file 12866_2024_3215_MOESM2_ESM.docx]

**Table S1:** The distribution of the resistance genes (*blaKPC, blaOXA-48, blaNDM, blaVIM, mcr1-mcr5*) among each isolate:

| The Number of apparent healthy camels’ samples | *mcr-I* | mcr-2 | *mcr-3* | *mcr-4* | *mcr-5* | bla_NDM_ | bla_OXA-48_ | bla_KPC_ | bla_VIM_ |
| --- | --- | --- | --- | --- | --- | --- | --- | --- | --- |
| 2 |  |  | +VE |  |  |  |  |  |  |
| 7 |  |  |  | +VE |  | +VE |  |  |  |
| 11 |  |  | +VE |  |  |  | +VE |  |  |
| 19 |  |  | +VE |  |  |  | +VE |  |  |
| 20 |  |  | +VE |  |  |  | +VE |  |  |
| 22 |  |  |  |  |  |  | +VE |  |  |
| 24 |  |  |  |  |  | +VE |  |  |  |
| 25 |  |  |  |  |  | +VE |  |  |  |
| 29 |  |  |  | +VE |  |  |  |  |  |
| 37 |  |  | +VE |  |  |  | +VE |  | +VE |
| 39 |  |  | +VE |  |  |  |  |  |  |
| 41 |  |  | +VE |  |  |  |  |  |  |
| 43 |  |  | +VE |  |  | +VE |  |  |  |
| 45 |  |  |  |  |  | +VE |  |  |  |
| 46 |  |  | +VE |  |  |  |  |  |  |
| 47 |  |  |  |  |  | +VE |  |  |  |
| 49 | +VE |  | +VE |  |  | +VE |  |  |  |
| 50 |  |  |  |  |  | +VE |  |  |  |
| 51 |  |  | +VE |  |  | +VE | +VE |  | +VE |
| 52 | +VE | +VE | +VE |  |  |  | +VE |  | +VE |
| 63 |  | +VE |  |  |  |  |  |  |  |
| 64 |  |  | +VE |  |  |  |  |  |  |
| 65 |  |  |  |  |  | +VE |  |  |  |
| 70 | +VE |  | +VE |  |  |  |  |  |  |
| 72 |  |  | +VE |  |  | +VE |  |  |  |
| 76 |  |  |  |  |  | +VE |  |  |  |
| 80 |  |  |  |  |  | +VE |  |  | +VE |
| 83 |  |  | +VE |  |  | +VE |  |  | +VE |
| 88 |  |  | +VE |  |  |  |  |  |  |
| 100 |  |  |  |  |  |  |  |  | +VE |
| 104 |  |  | +VE |  |  |  |  |  |  |
| 106 |  |  | +VE |  |  |  |  |  |  |
| 107 |  |  | +VE | +VE |  |  |  |  |  |
| 117 |  |  | +VE |  |  |  |  |  |  |
